# Reference genome of the kidnapper ant, *Polyergus mexicanus*

**DOI:** 10.1093/jhered/esae047

**Published:** 2024-09-09

**Authors:** Elizabeth I Cash, Merly Escalona, Philip S Ward, Ruta Sahasrabudhe, Courtney Miller, Erin Toffelmier, Colin Fairbairn, William Seligmann, H Bradley Shaffer, Neil D Tsutsui

**Affiliations:** Department of Environmental Science, Policy, and Management, University of California, Berkeley, Berkeley, CA, United States; Department of Environmental Engineering Sciences, University of Florida, Gainesville, FL, United States; Department of Biomolecular Engineering, University of California, Santa Cruz, Santa Cruz, CA, United States; Department of Entomology and Nematology, University of California, Davis, Davis, CA, United States; DNA Technologies and Expression Analysis Cores, University of California, Davis, Davis, CA, United States; La Kretz Center for California Conservation Science, Institute of the Environment and Sustainability, University of California, Los Angeles, CA, United States; La Kretz Center for California Conservation Science, Institute of the Environment and Sustainability, University of California, Los Angeles, CA, United States; Department of Ecology and Evolutionary Biology, University of California, Los Angeles, CA, United States; Department of Ecology and Evolutionary Biology, University of California, Santa Cruz, Santa Cruz, CA, United States; Department of Ecology and Evolutionary Biology, University of California, Santa Cruz, Santa Cruz, CA, United States; La Kretz Center for California Conservation Science, Institute of the Environment and Sustainability, University of California, Los Angeles, CA, United States; Department of Ecology and Evolutionary Biology, University of California, Los Angeles, CA, United States; Department of Environmental Science, Policy, and Management, University of California, Berkeley, Berkeley, CA, United States

**Keywords:** California Conservation Genomics Project, dulosis, Formicidae, *Formica*, host race, social parasite

## Abstract

*Polyergus* kidnapper ants are widely distributed, but relatively uncommon, throughout the Holarctic, spanning an elevational range from sea level to over 3,000 m. These species are well known for their obligate social parasitism with various *Formica* ant species, which they kidnap in dramatic, highly coordinated raids. Kidnapped *Formica* larvae and pupae become integrated into the *Polyergus* colony where they develop into adults and perform nearly all of the necessary colony tasks for the benefit of their captors. In California, *Polyergus mexicanus* is the most widely distributed *Polyergus*, but recent evidence has identified substantial genetic polymorphism within this species, including genetically divergent lineages associated with the use of different *Formica* host species. Given its unique behavior and genetic diversity, *P. mexicanus* plays a critical role in maintaining ecosystem balance by influencing the population dynamics and genetic diversity of its host ant species, *Formica*, highlighting its conservation value and importance in the context of biodiversity preservation. Here, we present a high-quality genome assembly of *P. mexicanus* from a sample collected in Plumas County, CA, United States, in the foothills of the central Sierra Nevada. This genome assembly consists of 364 scaffolds spanning 252.31 Mb, with contig N50 of 481,250 kb, scaffold N50 of 10.36 Mb, and Benchmarking Universal Single-Copy Orthologs (BUSCO) completeness of 95.4%. We also assembled the genome of the *Wolbachia* endosymbiont of *P. mexicanus*—a single, circular contig spanning 1.23 Mb. These genome sequences provide essential resources for future studies of conservation genetics, population genetics, speciation, and behavioral ecology in this charismatic social insect.

## Introduction

*Polyergus* kidnapper ants are obligate social parasites that are widespread, but relatively uncommon, in the northern hemisphere. In California, two nominal taxa are currently recognized, *Polyergus mexicanus* and *Polyergus vinosus*, and they parasitize several different species of ants from the closely related genus, *Formica*, by capturing larvae and pupae in dramatic, well-organized raids on *Formica* colonies (Trager 2013). The captured *Formica* mature into adult workers within the *Polyergus* colony, imprint on the colony odors in their new home, and begin to perform normal worker behaviors for the benefit of their *Polyergus* captors. As a result, foraging, nest excavation, brood care, and other essential tasks in *Polyergus* colonies are performed by their *Formica* captives (Topoff et al. 1989; Trager 2013). In California, *P. mexicanus* can parasitize at least eight out of an estimated 40 nominal *Formica* species present in the state, but each *Polyergus* colony usually specializes in using only one of these potential host species (Wheeler 1915; Ward 2005; Torres et al. 2018, Fig. 1A and B). Recent genetic studies, based on mitochondrial DNA and microsatellite markers, have shown that substantial cryptic diversity exists within *P. mexicanus*, where new host-associated races of *P. mexicanus* appear to be diverging into incipient species as they adapt to exploiting different *Formica* hosts (Torres et al. 2018). As greater clarity on this cryptic, co-evolutionary diversity unfolds, genomic resources including reference genomes and associated statewide population genomic sampling as part of the California Conservation Genomics Projects (CCGP; Shaffer et al. 2022) will help identify genetically unique populations, cryptic species, and geographic regions of high conservation value (Fiedler et al. 2022).

**Fig. 1.**
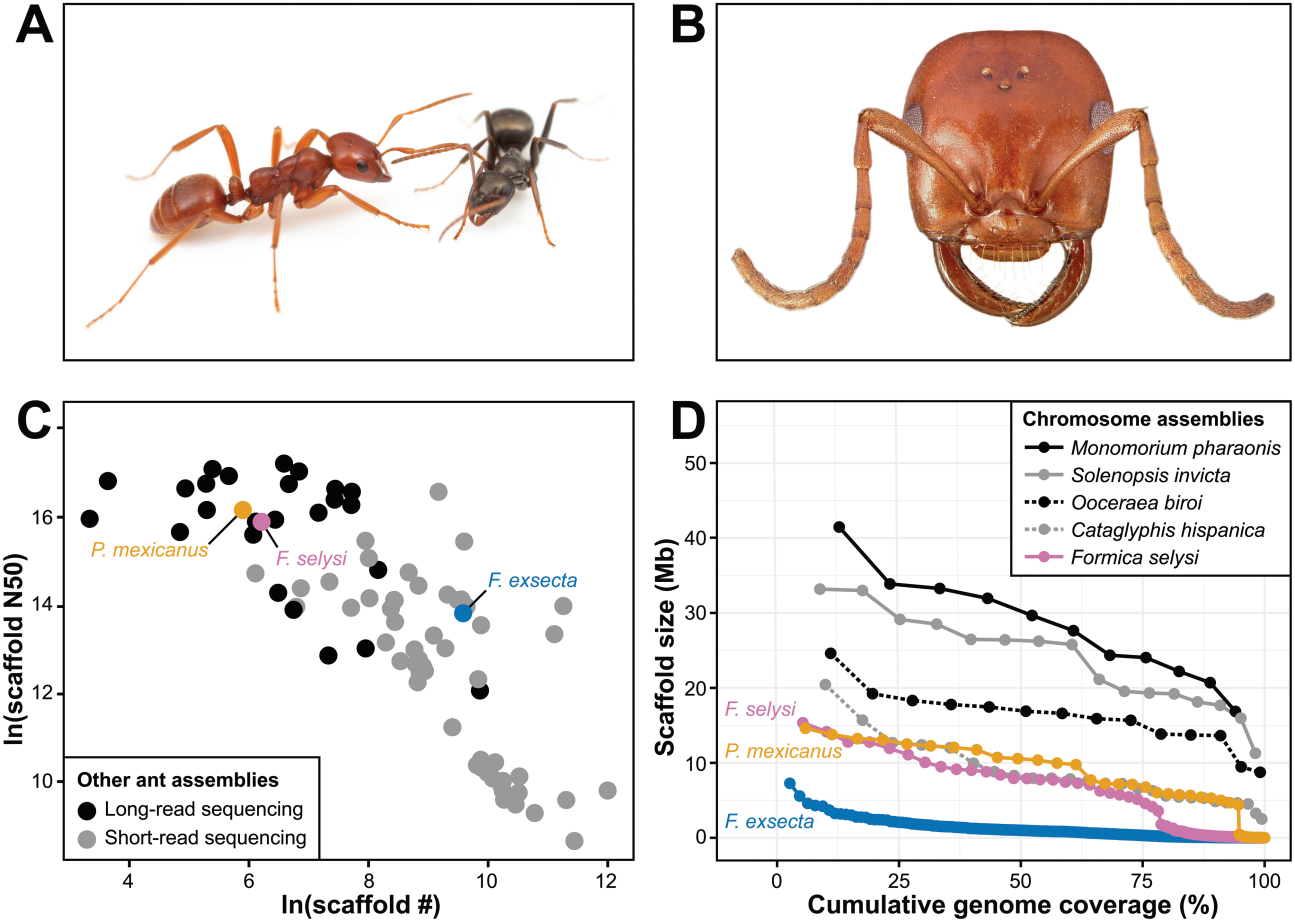
Kidnapper ant, *Polyergus mexicanus*, and genome assembly comparisons. A) Representative worker of *P. mexicanus* (left) with a parasitized host *Formica* worker (right) (image credit: Elizabeth I. Cash). B) Close-up of a *P. mexicanus* worker head showing distinctive sickle-shaped mandibles specialized for carrying raided larvae and pupae from host ant colonies (image credit: April Nobile, specimen: CASENT0005397, from: https://www.antweb.org). C) Scatterplot of genome metrics for 83 ant genomes representing 61 species with scaffold-level assemblies accessed via NCBI Datasets (see Supplementary Table S1 for full details). Three focal species, *P. mexicanus* (this study, long-read sequencing), *Formica selysi* (Brelsford et al. 2020, long-read sequencing), and *F. exsecta* (Dhaygude et al. 2019, short-read sequencing), are highlighted to compare scaffold assemblies of these closely related taxa with 80 other ant genome assemblies (shaded according to sequencing method, i.e. long- versus short-read lengths). D) Lineplot of genome metrics comparing six ant species with *P. mexicanus*. The size of each chromosome or scaffold (Mb) is displayed on the *y* axis and color formatted by species. The cumulative genome coverage (%) is represented on the *x* axis by the sum of the preceding chromosome or scaffold size(s) up to a given point. Chromosome-level assemblies for five ant species (*Cataglyphis hispanica*, *F. selysi*, *Monomorium pharaonis*, *Ooceraea biroi*, and *Solenopsis invicta*) are compared to focal ant species with scaffold-level assemblies (*P. mexicanus* and *F. exsecta*) showing the similarity between assembled scaffold sizes of *P. mexicanus* (this study) and chromosome sizes of a closely related species, *F. selysi* (see Supplementary Table S2).

Several features of *P. mexicanus* make it an important conservation target. First, the highly specialized life history of *P. mexicanus* translates into relative rarity, as this social parasite relies on thriving populations of its host *Formica* species to persist. In addition, the range of *P. mexicanus* extends to extremely high elevation sites throughout the Sierra Nevada and other mountain ranges in western United States, which are among the habitats likely to be affected by increasing global temperatures, changing patterns of precipitation, and increased frequency and intensity of wildfires. Finally, the extensive population differentiation and cryptic species diversity in this complex suggests that incipient species with highly restricted geographic ranges are likely to exist, and maybe imperiled by changing land use practices, habitat fragmentation, and global climate change. In this context, the CCGP is particularly interested in understanding and mitigating these risks through genetically informed conservation strategies (Shaffer et al. 2022).

We report here a de novo reference genome assembly for *P. mexicanus*, which serves as an important tool for analyzing population genomic datasets including divergence patterns within *P. mexicanus*. The reference genome also enables studies of the potential impacts of climate change on *P. mexicanus* at its range limits and offers insights into the evolution of their unique behaviors. Specifically, the genome allows researchers to investigate genetic adaptations related to thermal tolerance and stress responses (Franks and Hoffman 2012), as well as the genetic basis of parasitic behaviors due to loss of worker-associated genes, the gain of parasite-associated genes, and/or the reconfiguration of genes associated with social behavior (Cini et al. 2015; Smith et al. 2015), providing a comprehensive tool for understanding both ecological impacts and evolutionary mechanisms. Existing genomic resources for *Polyergus* include lower coverage double digest restriction-site associated DNA (ddRAD) sequences from a *P. mexicanus* male collected at Donner Pass in the Sierra Nevada and from a *P. vinosus* female collected from Santa Cruz Island, California (Brelsford et al. 2020). Additionally, genomic resources for closely related *Formica* species include a chromosome-level whole genome assembly of *F. selysi* (Brelsford et al. 2020) and a scaffold-level whole genome assembly of *F. exsecta* (Dhaygude et al. 2019). We highlight comparisons between *P. mexicanus* (this study) and the whole genome sequences of these two *Formica* species as well as the whole genome sequences of other ant species currently available in the NCBI genome database (Fig. 1C and D).

## Methods

### Biological materials

A large *P. mexicanus* colony fragment was collected from a fallen log approximately 2.8 km east of La Porte, California (39.69222, −120.95389) on 02 August 2020 by N. D. Tsutsui (collection number NDT 795). A single male alate (NDT 795.1) was used for HiFi SMRTbell library construction and sequencing and three adults (diploid female) workers (NDT 795.3) were used for Omni-C library construction and sequencing. Additionally, morphological analysis identified the host species of this colony as *Formica accreta* (determined by P. S. Ward, collection number NDT 795.H1). Voucher specimens from this colony were assigned unique specimen codes CASENT0885504 (*P. mexicanus*) and CASENT0885505 (host *F. accreta*), and have been deposited in the Bohart Museum of Entomology, University of California, Davis.

### High molecular weight genomic DNA isolation

A flash-frozen adult male ant was homogenized in 500 µL of homogenization buffer (10 mM Tris-HCL-pH 8.0 and 25 mM EDTA) using TissueRuptor II (Qiagen, Germany; Cat # 9002755). Lysis buffer (500 µL, 10 mM Tris, 25 mM EDTA, 200 mM NaCl, and 1% SDS) and proteinase K (500 µL, 100 µg/mL) were added to the homogenate, and it was incubated overnight at room temperature. Lysate was treated with RNAse A (20 µg/mL) at 37 °C for 30 min and was cleaned with equal volumes of phenol/chloroform using phase-lock gels (Quantabio Cat # 2302830). The DNA was precipitated by adding 0.4× volume of 5 M ammonium acetate and 3× volume of ice-cold ethanol. The DNA pellet was washed twice with 70% ethanol and resuspended in an elution buffer (10 mM Tris, pH 8.0). DNA was further cleaned with 1× KAPA Pure SPRI beads. Total DNA yield was 30 ng as measured by the Qubit 2.0 Fluorometer (Thermo Fisher Scientific, Waltham, MA). Integrity of the HMW gDNA was verified on a Femto pulse system (Agilent Technologies, Santa Clara, CA), with 52% of DNA in fragments larger than 50 kb in length.

### Nucleic acid library preparation

The HiFi SMRTbell library was constructed using the SMRTbell gDNA Sample Amplification Kit (Pacific Biosciences—PacBio, Menlo Park, CA; Cat. #101-980-000) and the SMRTbell Express Template Prep Kit 2.0 (PacBio; Cat. #100-938-900) according to the manufacturer’s instructions. HMW gDNA was sheared to around 10 kb using the Diagenode Megaruptor 3 system (Diagenode, Belgium; Cat. #B06010003). The sheared gDNA was incubated at 37 °C for 15 min to remove single-strand overhangs, followed by DNA damage repair at 37 °C for 30 min, end repair and A-tailing at 20 °C for 30 min and 65 °C for 30 min, and ligation of overhang adapters at 20 °C for 60 min. To prepare for library amplification by PCR, the library was purified with ProNex beads (Promega, Madison, WI; Cat. # NG2002) for two PCR amplification conditions at 15 cycles each then another ProNex beads purification. Purified amplified DNA from both reactions were pooled in equal mass quantities for another round of enzymatic steps that included DNA repair, end repair/A-tailing, overhang adapter ligation, and purification with ProNex Beads. The PippinHT system (Sage Science, Beverly, MA; Cat # HPE7510) was used for SMRTbell library size selection to remove fragments <6 kb. The 10 kb average HiFi SMRTbell library was sequenced at UC Davis DNA Technologies Core (Davis, CA) using one 8 M Single-molecule Real Time (SMRT) cell, Sequel II sequencing chemistry 2.0, and 30-h movies each on a PacBio Sequel II sequencer.

### Omni-C preparation

The Omni-C library was prepared using the Dovetail Omni-C Kit (Dovetail Genomics, Scotts Valley, CA) according to the manufacturer’s protocol with slight modifications. First, specimen tissue (whole individuals, three adult workers, NDT 795.3) was thoroughly ground with a mortar and pestle while cooled with liquid nitrogen. Subsequently, chromatin was fixed in place in the nucleus. The suspended chromatin solution was then passed through 100 µm and 40 µm cell strainers to remove large debris. Fixed chromatin was digested under various conditions of DNase I until a suitable fragment length distribution of DNA molecules was obtained. Chromatin ends were repaired and ligated to a biotinylated bridge adapter followed by proximity ligation of adapter-containing ends. After proximity ligation, crosslinks were reversed and the DNA was purified from proteins. Purified DNA was treated to remove biotin that was not internal to ligated fragments. An NGS library was generated using an NEB Ultra II DNA Library Prep kit (NEB, Ipswich, MA) with an Illumina-compatible y-adaptor. Biotin-containing fragments were then captured using streptavidin beads. The post-capture product was split into two replicates before PCR enrichment to preserve library complexity with each replicate receiving unique dual indices. The library was sequenced at Vincent J. Coates Genomics Sequencing Lab (Berkeley, CA) on an Illumina NovaSeq 6000 platform (Illumina, San Diego, CA) to generate approximately 100 million 2 × 150 bp read pairs per GB of genome length.

### DNA sequencing and genome assembly

#### Nuclear genome assembly

We assembled the *P. mexicanus* genome following the CCGP assembly pipeline for haploid species, as outlined in Table 1, which lists the tools and non-default parameters used in the assembly. The pipeline uses both the PacBio HiFi reads and Omni-C data to produce highly contiguous genome assemblies. First, we removed the remnant adapter sequences from the PacBio HiFi dataset using HiFiAdapterFilt (Sim et al. 2022) and generated an initial haploid assembly using HiFiasm (Cheng et al. 2022) based on the filtered PacBio HiFi reads. During the initial assembly, we specified no purging and the ploidy corresponding to the sequenced haploid male. From the generated output, we kept the file corresponding to the primary assembly file. We then aligned the Omni-C data to the assembly following the Arima Genomics Mapping Pipeline (https://github.com/ArimaGenomics/mapping_pipeline) and then scaffolded it with SALSA (Ghurye et al. 2017, 2019).

**Table 1.** Assembly pipeline and software used.

Assembly	Software and any non-default options	Version
Filtering PacBio HiFi adapters	HiFiAdapterFilt	Commit 64d1c7b
K-mer counting	Meryl (*k* = 21)	1
Estimation of genome size and heterozygosity	GenomeScope	2
De novo assembly (contiging)	HiFiasm (--n-hap 1 -l0)	0.16.1-r375
Scaffolding		
Omni-C data alignment	Arima Genomics Mapping Pipeline	Commit 2e74ea4
Omni-C Scaffolding	SALSA (-DNASE, -i 20, -p yes)	2
Gap closing	YAGCloser (-mins 2 -f 20 -mcc 2 -prt 0.25 -eft 0.2 -pld 0.2)	Commit 0e34c3b
Omni-C Contact map generation		
Short-read alignment	BWA-MEM (-5SP)	0.7.17-r1188
SAM/BAM processing	samtools	1.11
SAM/BAM filtering	pairtools	0.3.0
Pairs indexing	pairix	0.3.7
Matrix generation	cooler	0.8.10
Matrix balancing	hicExplorer (hicCorrectmatrix correct --filterThreshold -2 4)	3.6
Contact map visualization	HiGlass	2.1.11
	PretextMap	0.1.4
	PretextView	0.1.5
	PretextSnapshot	0.0.3
Manual curation tools	Rapid curation pipeline (Wellcome Trust Sanger Institute, Genome Reference Informatics Team)	Commit 4ddca450
Genome quality assessment		
Basic assembly metrics	QUAST (--est-ref-size)	5.0.2
Assembly completeness	BUSCO (-m geno, -l hymenoptera)	5.0.0
	Merqury	2020-01-29
Contamination screening		
Local alignment tool	BLAST+ (-db nt, -outfmt “6 qseqid staxids bitscore std,” -max_target_seqs 1, -max_hsps 1, -evalue 1e-25)	2.10
General contamination screening	BlobToolKit (PacBIo HiFi Coverage, NCBI Taxa ID = 262038, BUSCODB = Hymenoptera)	2.3.3
Mitochondrial assembly		
Mitochondrial genome assembly	MitoHiFi (-r, -p 80, -o 1 -a animal)	2.2
*Wolbachia* assembly		
Sequence alignment	lastz (--nogapped,--notransition, –step = 20)	1.04.15
Alignment visualization	LAJ (http://globin.cse.psu.edu/dist/laj/)	2005-12-14
Local alignment tool	BLAST+ (-db nt, -outfmt “6 qseqid staxids bitscore std,” -max_target_seqs 1, -max_hsps 1, -evalue 1e-25)	2.1
Completeness assessment	CheckM (taxon sets: genus *Wolbachia*, family *Anaplasmataceae*)	1.2.2
Genome annotation	bakta (https://bakta.computational.bio/)	1.9.1| DB: 5.0.0

Software citations are listed in the text.

The assembly was manually curated by generating and analyzing the corresponding Omni-C contact maps and breaking scaffolds when misassemblies were identified. In general, to generate the contact maps, we aligned the Omni-C data with BWA-MEM (Li 2013), identified ligation junctions, and generated Omni-C pairs using pairtools (Open2C et al. 2024). We generated multi-resolution Omni-C matrices with cooler (Abdennur and Mirny 2020) and balanced them with hicExplorer (Ramírez et al. 2018). We used HiGlass (Kerpedjiev et al. 2018) and the PretextSuite (https://github.com/wtsi-hpag/PretextView; https://github.com/wtsi-hpag/PretextMap; https://github.com/wtsi-hpag/PretextSnapshot) to visualize the contact maps where we identified misassemblies and misjoins. Some remaining gaps (joins generated during scaffolding and/or curation) were closed using the PacBio HiFi reads and YAGCloser (https://github.com/merlyescalona/yagcloser). Finally, we checked for contamination using the BlobToolKit Framework (Challis et al. 2020).

### Genome quality assessment

We generated k-mer counts from the PacBio HiFi reads using meryl (https://github.com/marbl/meryl). The k-mer counts were then used in GenomeScope 2.0 (Ranallo-Benavidez et al. 2020) to estimate genome features including genome size, heterozygosity, and repeat content. To obtain general contiguity metrics, we ran QUAST (Gurevich et al. 2013). To evaluate genome quality and functional completeness, we used Benchmarking Universal Single-Copy Orthologs (BUSCO) (Manni et al. 2021) with the Hymenoptera ortholog database (hymenoptera_odb10) which contains 5,991 genes. Base level accuracy (QV) and k-mer completeness were assessed using the previously generated meryl database and merqury (Rhie et al. 2020). We further estimated genome assembly accuracy via BUSCO gene set frameshift analysis using the pipeline described in Korlach et al. (2017). Given that the specimen used for the assembly is haploid, measurements of the size of the phased blocks are based on the size of the final contigs. We follow the quality metric nomenclature established by Rhie et al. (2021), with the genome quality code *x*.*y*.*P*.*Q*.*C*, where, *x* = log10[contig NG50]; *y* = log10[scaffold NG50]; *P* = log10 [phased block NG50]; *Q* = Phred base accuracy QV (quality value); *C* = % genome represented by the first “*n*” scaffolds, assuming a karyotype of *n* = 27, which is the number of chromosomes for other species in the genus (Imai 1966).

### Mitochondrial genome assembly

We assembled the *P. mexicanus* mitochondrial genome from the PacBio HiFi reads using the reference-guided pipeline MitoHiFi (Allio et al. 2020; Uliano-Silva et al. 2023). The mitochondrial sequence of *Formica sinae* (NCBI: NC_060873.1) was used as the starting sequence. After completion of the nuclear genome, we searched for matches of the resulting mitochondrial assembly sequence in the nuclear genome assembly using BLAST+ (Camacho et al. 2009) and filtered out contigs and scaffolds from the nuclear genome with a sequence identity >99% and size smaller than the mitochondrial assembly sequence. No other manual curation was performed on the mitochondrial genome.

### Endosymbiont genome assembly

We used the genome of *Wolbachia* endosymbiont of *Cardiocondyla obscurior* (NCBI: GCA_902713645.1; WoCaobscurior_wCobs-JP2010-OypB_1) as a guide to find the *Wolbachia* endosymbiont genome present in our initial *P. mexicanus* assembly. We aligned the contigs removed from the nuclear genome in the contamination process to the assembly ID reference using BLAST+. We visually inspected the circularity of the endosymbiont contig using lastz (Harris 2007) and LAJ (Wilson et al. 2001). Finally, we used bakta (Schwengers et al. 2021) to generate a draft genome annotation of the bacterial genome and we used CheckM (Parks et al. 2015) to assess completeness of the genome.

### Genome assembly comparisons

We compared basic scaffold-level genome assembly metrics for 61 ant species currently available in the NCBI genome database (Supplementary Table S1). Scaffold number versus scaffold N50 (ln transformed) were plotted using ggplot2 in R (Wickham 2016) to visualize differences in contiguity between ant genome assemblies (Fig. 1C). Additionally, scaffold and chromosome sizes (Mb) were plotted relative to genome coverage (%) for four ant species with chromosome-level assemblies (*Cataglyphis hispanica*, *Monomorium pharaonis*, *Ooceraea biroi*, and *Solenopsis invicta*) along with two *Formica* species, *F. exsecta* (Dhaygude et al. 2019) and *F. selysi* (Brelsford et al. 2020), and the kidnapper ant, *P. mexicanus* (this study), to compare mapping results among genome assemblies (Fig. 1D, Supplementary Table S2).

## Results

### Sequencing data

**Fig. 2.**
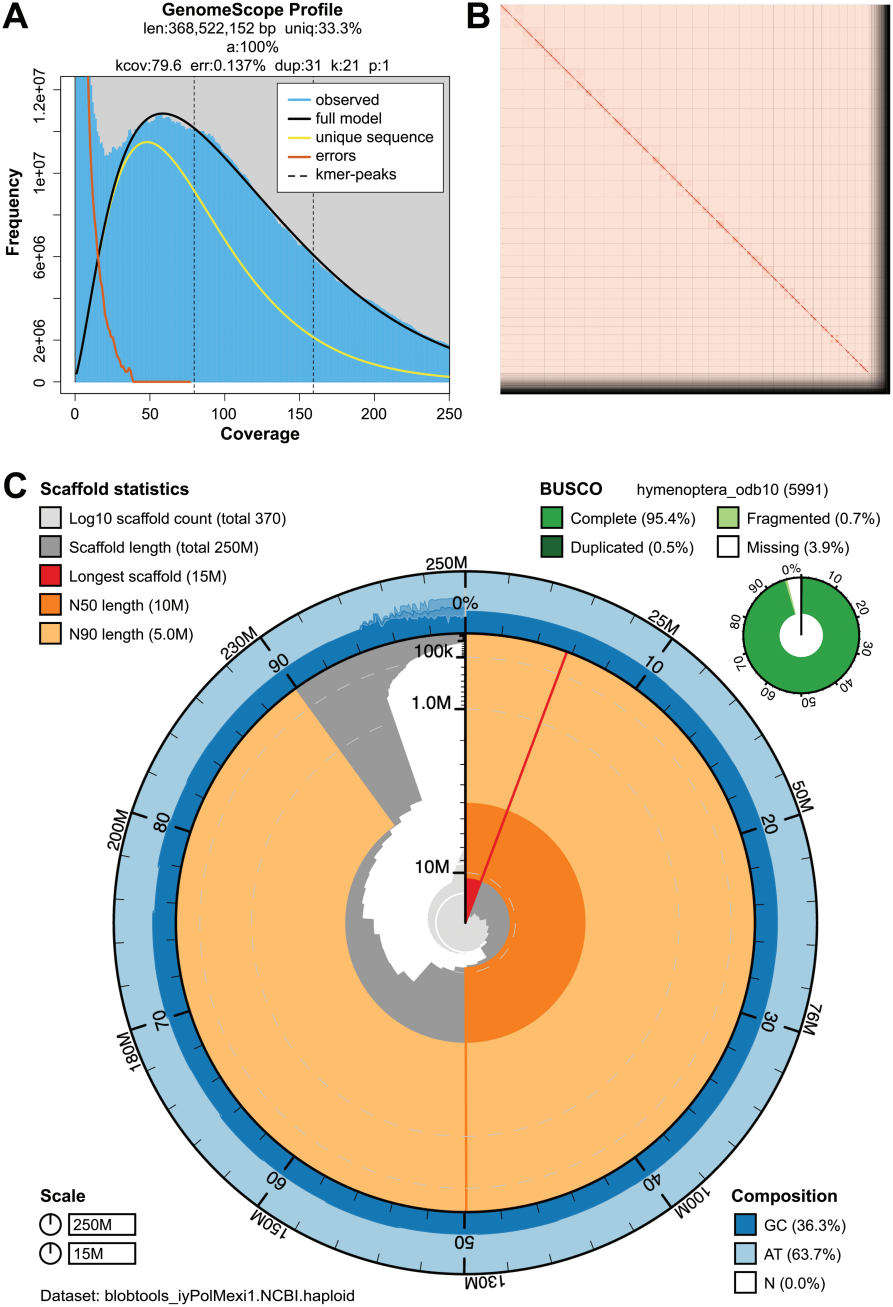
Visual overview of *Polyergus mexicanus* genome assembly metrics. A) K-mer spectra output generated from PacBio HiFi data without adapters using GenomeScope 2.0. The observed unimodal pattern corresponds to a haploid genome. B) Omni-C contact map for the scaffold-level genome assembly generated with PretextSnapshot. The Omni-C contact map translates proximity of genomic regions in 3D space to contiguous linear organization. Each cell in the contact map corresponds to sequencing data supporting the linkage (or join) between two such regions. Scaffolds are separated by black lines, with higher density corresponding to higher levels of fragmentation. C) BlobToolKit Snail plot showing a graphical representation of the quality metrics presented in Table 2 for the *P. mexicanus* assembly (iyPolMexi1) and BUSCO assessment results based on the Hymenoptera set of orthologous genes (*n* = 5,991). The plot circle represents the full size of the assembly. From the inside to the outside, the central plot covers length-related metrics. The red line represents the size of the longest scaffold; all other scaffolds are arranged in size-order moving clockwise around the plot and drawn in gray starting from the outside of the central plot. Dark and light orange arcs show the scaffold N50 and scaffold N90 values, respectively. The central light gray spiral shows the cumulative scaffold count with a white line at each order of magnitude. White regions in this area reflect the proportion of Ns in the assembly. The dark versus light blue area around it shows mean, maximum, and minimum GC versus AT content at 0.1% intervals.

The Omni-C and PacBio HiFi sequencing libraries generated 102.99 million read pairs and 3.25 million reads, respectively. The latter yielded 101.03-fold coverage (N50 read length 9,167 bp; minimum read length 123 bp; mean read length 9,181 bp; maximum read length of 33,305 bp) based on the GenomeScope 2.0 genome size estimation of 368.52 Mb. We estimated a 0.137% sequencing error based on PacBio HiFi reads, and the k-mer spectrum shows a unimodal distribution with a single major peak at ~80 (Fig. 2A).

**Table 2.** Sequencing and assembly statistics, and accession numbers.

Bio projects and vouchers						
CCGP NCBI BioProject		PRJNA720569				
Genera NCBI BioProject		PRJNA765828				
Species NCBI BioProject		PRJNA808362				
NCBI BioSample		SAMN35821861, SAMN35821862				
Specimen identification		NDT 795.1, NDT 795.3				
NCBI Genome accessions						
Assembly accession		GCA_030449975.1				
Genome sequences		JAUDSZ000000000				
Genome sequence						
PacBio HiFi reads	Run	1 PACBIO_SMRT (Sequel II) run: 3.3 M spots, 29.5 G bases, 14Gb				
	Accession	SRX21253684				
Omni-C Illumina reads	Run	2 ILLUMINA (Illumina NovaSeq 6000) runs: 103 M spots, 31.1 G bases, 10.2 Gb				
	Accession	SRX21253685, SRX21253686				
Genome Assembly Quality Metrics						
Assembly identifier (Quality code^a^)		iyPolMexi1(5.6.P5.Q55.C95)				
HiFi Read coverage^b^		101.03×				
Number of contigs		1,151				
Contig N50 (bp)		481,250				
Contig NG50^b^		399,645				
Longest Contigs		3,834,916				
Number of scaffolds		364				
Scaffold N50		10,364,369				
Scaffold NG50^b^		9,764,453				
Largest scaffold		14,608,128				
Size of final assembly (bp)		252,309,906				
Phased block NG50^b^		419,568				
Gaps per Gbp (#Gaps)		3121(788)				
Indel QV (Frame shift)		43.65515337				
Base pair QV		55.3601				
k-mer completeness		96.154				
BUSCO completeness^c^ (Hymenoptera) *n* = 5,991		**C**	**S**	**D**	**F**	**M**
		95.40%	94.90%	0.50%	0.70%	3.90%
Organelles (partial mitochondrial sequence)	Size (bp)	15,803				
	Accession	JAUDSZ010000365.1				
Endosymbionts (*Wolbachia* sequence)	Size (bp)	1,225,351				
	Completeness^d^	98.55%				
	Accession	CP158586				

^a^Assembly quality code *x*.*y*.*P*.*Q*.*C* derived notation, from Rhie et al. (2021). *x* = log10[contig NG50]; *y* = log10[scaffold NG50]; *P* = log10 [phased block NG50]; *Q* = Phred base accuracy QV (Quality value); *C* = % genome represented by the first “*n*” scaffolds, following a known karyotype for *Polyergus samurai* of *n* = 27 (Imai 1966).

^b^Read coverage and NGx statistics have been calculated based on the estimated genome size of 368.52 Mb.

^c^BUSCO Scores. Complete BUSCOs (C). Complete and single-copy BUSCOs (S). Complete and duplicated BUSCOs (D). Fragmented BUSCOs (F). Missing BUSCOs (M).

^d^Completeness (%) assessed with *Wolbachia* CheckM marker set.

### Nuclear and mitochondrial genome assembly

The size of the final *P. mexicanus* assembly (iyPolMexi1) was similar but not equal to the estimated value from GenomeScope 2.0 (Fig. 2A), as has been observed in other taxa (see Pflug et al. 2020 for an example). The genome coverage was 101.03× and the assembly consists of 364 scaffolds spanning 252.31 Mb, with contig N50 of 0.48 Mb, scaffold N50 of 10.36 Mb, largest contig of 3.83 Mb, and largest scaffold of 14.60 Mb. The estimated BUSCO completeness score of the iyPolMexi1 assembly corresponds to 95.4% using the Hymenoptera gene set, with a per base quality (QV) of 55.36, a k-mer completeness of 96.15%, and a frameshift indel QV of 43.65.

During manual curation, we made 461 joins and 34 breaks. In the gap-closing step, we were able to close a total of three gaps. We filtered out 91 contigs corresponding to bacterial contamination from *Fructilactobacillus* (2,579,852 bp), *Oecophyllibacter* (2,314,524 bp), *Wolbachia* (1,603,876 bp), and an undefined *Acetobacteraceae* bacterium (2,078,418 bp), as well as two contigs corresponding to viral contamination from *Iridovirus* Liz-CrIV (192,140 bp, Supplementary Table S3). No further contigs were removed. The Omni-C contact map of the scaffold assembly shows that the genome was highly contiguous (Fig. 2B). A graphical representation of the genome assembly is shown in Fig. 2C, and detailed assembly statistics are reported in Table 2. We have deposited the genome assembly in GenBank (see Table 2 and Data Availability for details).

We assembled a partial mitochondrial genome for *P. mexicanus* with MitoHiFi. The mitochondrial sequence has a size of 15,803 bp, with a nucleotide composition biased toward *A* + *T* content (*A* = 40.02%, *T* = 41.38%, *G* = 12.90%, *C* = 5.69%), and consists of 11 unique transfer RNAs and 17 protein-coding genes. Given its smaller, potentially incomplete, size (i.e. 90.7% of the bp length compared with *Formica sinae*), the partial mitochondrial assembly is included with the nuclear genome assembly as a separate scaffold (Table 2).

### Endosymbiont genome assembly

The final *Wolbachia* endosymbiont of the *P. mexicanus* genome (icPolMex1_wolbachia, GenBank accession CP158586) was a single, circular, gapless contig with a final size of 1,225,351 bp (Supplementary Fig. S1), which was similar to the reference used as a guide (*Wolbachia* endosymbiont of *C. obscurior*, GCA_902713645.1; genome size = 1,299,373 bp, Supplementary Table S4). CheckM analysis of genome completeness at the genus level (*Wolbachia*) and the family level (*Anaplasmataceae*) were 98.55% and 99.06%, respectively, and the contamination and strain heterogeneity values were 0.00% for both taxonomic-level comparisons. The base composition of the final assembly was *A* = 32.38%, *T* = 32.28%, *G* = 17.77%, *C* = 17.57%. Bakta annotation of the *Wolbachia* genome identified 1,161 coding sequences, 34 transfer RNAs, 1 transfer-messenger RNA, 3 ribosomal RNAs, 18 non-coding RNAs, 1 non-coding RNA cis-regulatory region, and 1 origin of replication (*oriC*) feature.

### Assembly comparisons

Genome metrics indicate that the kidnapper ant assembly was highly contiguous (364 scaffolds, scaffold N50 of 10.36 Mb), with a scaffold number and scaffold N50 comparable to other available ant genomes generated with long-read sequencing methods (Fig. 1C, Supplementary Table S1). Although chromosome assignments were not determined for *P. mexicanus*, 27 out of the 364 total scaffolds in the genome assembly approach sizes >4 Mb (mean ± SD = 8.84 ± 3.26 Mb), make up >94% of the genome assembly, and are comparable to the average chromosome sizes of genome assemblies from five representative ant species including the closely related species *F. selysi* (mean ± SD = 8.41 ± 3.05 Mb, Fig. 1D, Supplementary Table S2).

## Discussion

The development of this reference genome for *P. mexicanus* fills an important gap for a genus that has a rich history of behavioral ecological research, but relatively few genomic resources. As obligate social parasites, *Polyergus* species exhibit a syndrome of unusual mating, colony-founding, and host-kidnapping behaviors (Trager 2013; Sapp et al. 2020). At the same time, these species have lost the ability to perform most typical worker ant behaviors, including foraging, nest excavation, and brood care (Topoff 1990). Persistence of *P. mexicanus* populations relies on robust populations of their host *Formica*, and recent studies in California have shown that *P. mexicanus* forms specialized, genetically divergent, host-associated lineages (Torres et al. 2018). In addition to their direct impacts on host *Formica* species, *Polyergus* are also influenced by host-competition with other socially parasitic *Formica* species and play important roles in maintaining ecosystem balance through these interactions (Mori et al. 2001). Currently, there are no known genomic underpinnings linked to the specific suite of parasitic behaviors exhibited by *Polyergus* kidnapper ants. The reference genome presented here provides a critical starting point for future studies into the genetic basis of this behavioral co-evolution.

Our *P. mexicanus* reference genome assembly is highly accurate, with excellent coverage (>100×) and BUSCO completeness (>95%). Compared to other ant genome assemblies, it is also highly contiguous with scaffold and scaffold N50 values similar to that of the chromosome-level genome assembly of the closely related *F. selysi* as well as other ant species sequenced with long-read methods (Fig. 1C, Supplementary Table S1). The 27 largest *P. mexicanus* scaffolds comprise 94.6% of the genome assembly, matching the predicted chromosome number of *n* = 27 for *P. mexicanus* based on the reported karyotypes of the related species *Polyergus samurai* (Imai 1966). Furthermore, these 27 scaffolds are similar to the assembled chromosome sizes of *F. selysi* and four other representative ant species (Fig. 1D, Supplementary Table S2). Taken together, these results indicate that our *P. mexicanus* genome is essentially a chromosome-level assembly, with less than 6% fragmented into smaller scaffolds.

The assembled size of this genome (252.31 Mb) is very close to genome size estimates by flow cytometry of *P. mexicanus* from Sagehen Creek, California (six individuals; mean = 262.9 Mb ± 1.3 SE) and *Polyergus breviceps* from near Barfoot Peak, Arizona (three individuals; mean = 264.1 Mb ± 2.7 SE, N. D. Tsutsui and J. S. Johnston, unpublished data). Although other *Polyergus* reference genomes have not been published, comparison to genomes of the relatively closely related *Formica* suggests that *Polyergus* may have reduced genome sizes. Our *P. mexicanus* assembly is moderately smaller than the assemblies for *F. exsecta* (277.7 Mb, Dhaygude et al. 2019) and *F. selysi* (290 Mb, Brelsford et al. 2020), and much smaller than the flow cytometry estimate for *F. pallidefulva* (385.1 Mb, Tsutsui et al. 2008). Our *P. mexicanus* mitochondrial genome assembly (15,803 bp) is also smaller than the mitochondrial genome assembly of *F. selysi* (16,752 bp, Brelsford et al. 2020), although it is currently unclear if the small size might be due to incomplete assembly or reduced genome size for this species. Despite this, the inclusion of the partial mitochondrial genome assembly is valuable for future studies of mitochondrial evolution in this genus. Development of additional genomic resources will reveal if reduced genome size is a general pattern across *Polyergus* and, if so, may indicate that the parasitic life history of *Polyergus* has led directly to genomic reductions.

We also assembled the *Wolbachia* bacterial endosymbiont of *P. mexicanus* whose size and completeness are similar to assemblies of *Wolbachia* endosymbionts reported for other ant species available on NCBI’s Genome database (Supplementary Table S4). *Wolbachia* endosymbionts play a significant role in insect biology, influencing aspects of reproduction, behavior, and immunity (Werren et al. 2008), and in ants *Wolbachia* have been shown to influence sex ratios, colony development, and nutrient metabolism (Pontieri et al. 2017; Cheng et al. 2019; Singh and Linksvayer 2020). Understanding the genomic mechanisms underlying *Wolbachia*–ant interactions can provide insights into ant evolution and the effects of *Wolbachia* on ant biodiversity. Furthermore, uncovering the diversity and distribution of *Wolbachia* strains in ants contributes to our broader understanding of microbial symbiosis and its impact on ecological communities.

Overall, the reference genome of the *P. mexicanus* kidnapper ant will be a crucially important resource for future studies of topics including speciation and the evolution of reproductive isolation, host/parasite co-evolution, behavioral ecology, and chemical ecology. In addition, this reference genome sequence will improve our understanding of conservation issues related to California *Polyergus*, which likely include geographically restricted California endemic species of this vulnerable group of ants (IUCN 2023). These studies will clarify the taxonomy of the species in this genus, contribute to larger goals of the CCGP (Shaffer et al. 2022), and fill an important phylogenetic gap in our genomic understanding of California biodiversity (Toffelmier et al. 2022).

## Supplementary material

Supplementary material is available at *Journal of Heredity* online.

esae047_suppl_Supplementary_Table_S1

esae047_suppl_Supplementary_Table_S2

esae047_suppl_Supplementary_Table_S3

esae047_suppl_Supplementary_Figure_S1

esae047_suppl_Supplementary_Table_S4

## Data Availability

Data generated for this study are available under NCBI BioProject PRJNA808362. Raw sequencing data for samples NDT 795.1 and NDT 795.3 (NCBI BioSamples SAMN35821861 and SAMN35821862) are deposited in the NCBI Short Read Archive (SRA) under SRX21253685 and SRX21253686 for Omni-C Illumina sequencing data, and SRX21253684 for the PacBio HiFi sequencing data. GenBank accession for the assembly is GCA_030449975.1; and for genome sequences JAUDSZ000000000. The GenBank accession for the mitochondrial sequence is JAUDSZ010000365.1 and for the Wolbachia endosymbiont sequence is CP158586. Assembly pipeline, scripts, and other data for the analyses presented can be found at the following GitHub repository: www.github.com/ccgproject/ccgp_assembly.
