# Supplementary material for: Reference genome of the kidnapper ant, Polyergus mexicanus
Source: J Hered. 2024 Sep 9;116(3):293–302. doi: 10.1093/jhered/esae047 (PMC12130431; doi:10.1093/jhered/esae047)
Supplement: esae047_suppl_Supplementary_Figure_S1 [file esae047_suppl_supplementary_figure_s1.pdf]

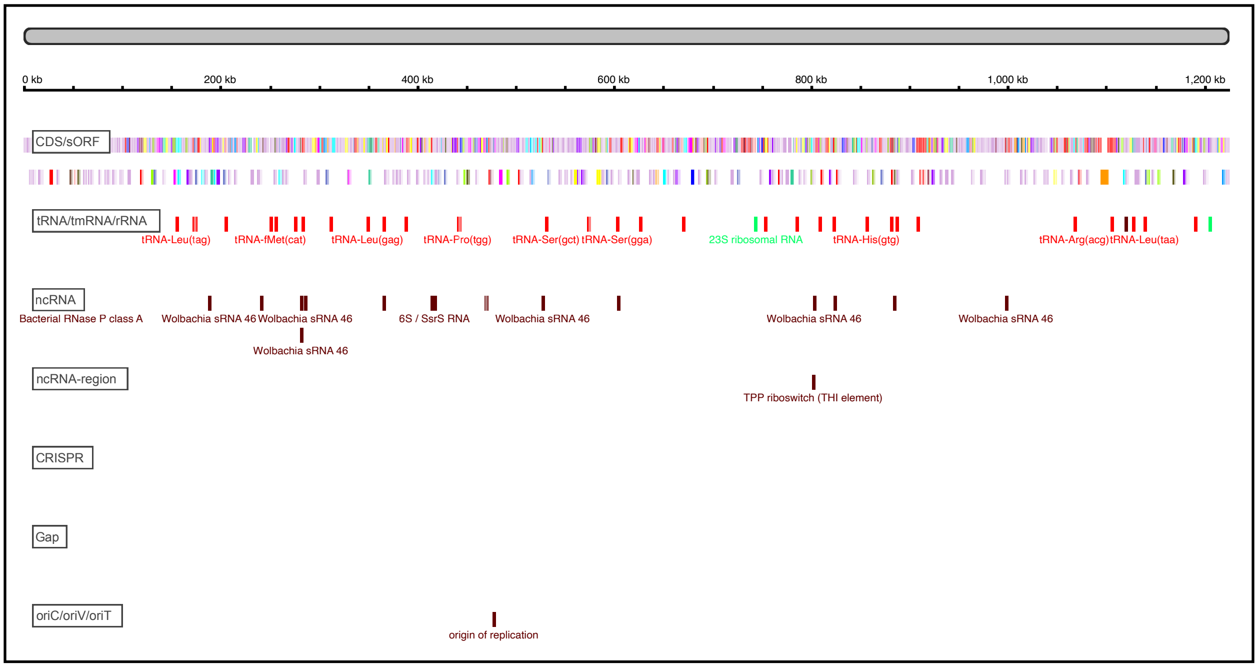

Figure S1. Linearized visualization of Bakta annotated genome features for the *Wolbachia* endosymbiont of *Polyergus mexicanus*. Coding sequence (CDS) and open reading frame (ORF) features are colored according to their functional category.
